# Intranasal Administration of Human MSC for Ischemic Brain Injury in the Mouse: *In Vitro* and *In Vivo* Neuroregenerative Functions

**DOI:** 10.1371/journal.pone.0112339

**Published:** 2014-11-14

**Authors:** Vanessa Donega, Cora H. Nijboer, Luca Braccioli, Ineke Slaper-Cortenbach, Annemieke Kavelaars, Frank van Bel, Cobi J. Heijnen

**Affiliations:** 1 Lab. of Neuroimmunology and Developmental Origins of Disease, University Medical Center Utrecht, Utrecht, The Netherlands; 2 Cell Therapy Facility, Dept. of Clinical Pharmacy, University Medical Center Utrecht; 3 Dept. of Symptom Research, MD Anderson Cancer Center, Houston TX, United States of America; 4 Dept. of Neonatology, University Medical Center Utrecht; Robert Debre Hospital, France

## Abstract

Intranasal treatment with C57BL/6 MSCs reduces lesion volume and improves motor and cognitive behavior in the neonatal hypoxic-ischemic (HI) mouse model. In this study, we investigated the potential of human MSCs (hMSCs) to treat HI brain injury in the neonatal mouse. Assessing the regenerative capacity of hMSCs is crucial for translation of our knowledge to the clinic. We determined the neuroregenerative potential of hMSCs *in vitro* and *in vivo* by intranasal administration 10 d post-HI in neonatal mice. HI was induced in P9 mouse pups. 1×10^6^ or 2×10^6^ hMSCs were administered intranasally 10 d post-HI. Motor behavior and lesion volume were measured 28 d post-HI. The *in vitro* capacity of hMSCs to induce differentiation of mouse neural stem cell (mNSC) was determined using a transwell co-culture differentiation assay. To determine which chemotactic factors may play a role in mediating migration of MSCs to the lesion, we performed a PCR array on 84 chemotactic factors 10 days following sham-operation, and at 10 and 17 days post-HI. Our results show that 2×10^6^ hMSCs decrease lesion volume, improve motor behavior, and reduce scar formation and microglia activity. Moreover, we demonstrate that the differentiation assay reflects the neuroregenerative potential of hMSCs *in vivo*, as hMSCs induce mNSCs to differentiate into neurons *in vitro*. We also provide evidence that the chemotactic factor CXCL10 may play an important role in hMSC migration to the lesion site. This is suggested by our finding that CXCL10 is significantly upregulated at 10 days following HI, but not at 17 days after HI, a time when MSCs no longer reach the lesion when given intranasally. The results described in this work also tempt us to contemplate hMSCs not only as a potential treatment option for neonatal encephalopathy, but also for a plethora of degenerative and traumatic injuries of the nervous system.

## Introduction

Neonatal encephalopathy due to perinatal hypoxia-ischemia (HI) is an important cause of mortality and long-term neurological deficits such as cerebral palsy, seizures and mental retardation in babies born at term [1]–[5]. However, therapeutic strategies for neonatal encephalopathy remain scarce. Hence, developing new treatment options for the newborn infant that effectively prevent or diminish the development of encephalopathy is of pivotal importance.

Bone marrow-derived mesenchymal stem/stromal cells (MSCs) have been shown to promote tissue repair in various disease models ranging from cardiovascular [6] to graft-versus-host disease [7]. MSCs are valuable as a therapeutic tool as they are hardly immunogenic due to a lack of MHC class II expression and co-stimulatory proteins (e.g. CD80, CD86 and CD40) [8], [9]. Therefore, several clinical trials are currently investigating the efficacy and safety of MSCs as a treatment option for various pathologies [10].

We have previously shown in a mouse model of neonatal HI brain damage that intranasal administration of *murine* MSCs significantly improves motor and cognitive behavior and reduces cerebral lesion volume [11]. In contrast to current pharmacological therapies for neonatal HI [12], we found that MSC treatment has a long therapeutic window of 10 days after the insult. Studies from our group and others have shown that intracranial and intravenous injection of murine MSCs actively promote proliferation and differentiation of neuronal and glial precursor cells as well as axonal regeneration [13]–[17]. Moreover, MSCs have been shown to exert strong anti-inflammatory properties and to modulate immune responses, for example by suppressing the proliferation of T cells and B cells in various disease models such as graft-versus-host disease [7], [18].

Before MSCs can be used in the clinic for the treatment of neonatal brain damage, the neuroregenerative potential of *human* MSCs (hMSCs) has to be determined. A few studies in the adult rodent MCAO model for stroke have investigated the efficacy of hMSCs to repair stroke induced brain lesion and behavioral deficits, but none have studied the effects of hMSCs on neonatal encephalopathy [19]–[21]. The results from these studies show that hMSCs improve motor behavior, decrease lesion size and enhance angiogenesis. In our study, we used an *in vitro* assay to assess the capacity of hMSCs to induce mouse neural stem cell (mNSC) to differentiate towards neuronal and glial cell fates. Moreover, we determined *in vivo* whether hMSCs are able to migrate towards the injury site in our mouse model of neonatal HI brain injury and which chemotactic factors may mediate MSC migration to the lesion. Most importantly, we investigated whether treatment with hMSCs improves motor behavior and decreases lesion size and gliosis following HI injury in the neonatal mice.

## Material and Methods

### Ethics statement, hMSCs isolation, culture and characterization

MSCs are classified as Advanced Therapy Medicinal Products and expanded in the GMP-accredited Cell Therapy Facility of the UMC Utrecht. Bone marrow from healthy donors is harvested for the expansion of MSCs as approved by the Dutch Central Committee on Research Involving Human Subjects (CCMO) (Biobanking bone marrow for MSC expansion, NL41015.041.12). Either the bone marrow donor or the parent or legal guardian of the donor signed the informed consent approved by the CCMO. The MSCs are isolated from the bone marrow by plastic adherence and expanded using platelet lysate. Optimal MSC expansion is achieved using human platelet lysate as substitute for fetal bovine serum in alpha-MEM (Macopharma, Utrecht, The Netherlands) [22]. Briefly, mononuclear cells from bone marrow are isolated using a density separation method. Isolation and expansion of MSCs is done by plastic adherence using 2-layer CellStacks in combination with Macopharma seeding sets, medium exchange sets and harvesting sets. MSCs are harvested using TripLE and Passage 3 MSCs are cryopreserved in bags in Physiological Salt solution containing human serum albumin and 10% DMSO for clinical trials. The bags are stored in the vapour phase of liquid nitrogen. After thawing, ±95% of the hMSCs are alive and positive for CD73, CD90 and CD105 and contain less than 0.1% CD-45 positive cells (Supplemental Figure 1). The hMSCs used in this study meet the release criteria set by the International Society for Cellular Therapy (i.e. more than 95% of the MSC are CD73, CD90 and CD105 positive) [23], [24]. Furthermore, the MSCs are sterile (negative for bacteria, yeast, funghi, mycoplasma (<10 CFU) and endotoxin (<5 EU/kg/hr)).

### Ethics statement, HI induction and intranasal MSC administration

Experiments were performed according to the Dutch and European international guidelines (Directive 86/609, ETS 123, Annex II) and approved by the Experimental Animal Committee Utrecht (University Utrecht, Utrecht, Netherlands). All efforts were made to minimize suffering.

An unilateral HI lesion was induced in 9 day old C57BL/6 mouse pups (Harlan Laboratories, Boxmeer, The Netherlands) under isoflurane anesthesia, by permanent occlusion of the right common carotid artery followed by hypoxia for 45 min at 10% oxygen. Control sham-operated mouse pups underwent anesthesia and incision only. The HI procedure resulted in a mortality rate of 10%. Pups from 11 litters were randomly assigned to the different experimental groups. Analyses were performed in a blinded set-up. At 10 days after HI, either 1×10^6^ or 2×10^6^ human MSCs (Passage 3) or PBS (vehicle-treatment) was administered intranasaly. 3 µl of hyaluronidase in PBS (100 U, Sigma-Aldrich, St. Louis, MO) was administered twice to each nostril to increase the permeability of the nasal mucosa. Thirty minutes later animals received 3 µL twice to each nostril with a total volume of 12 µL. We included an igloo and walking wheel in the cage as additional cage enrichment since it has been shown that physical exercise supports neurogenesis [25].

### *In vitro* proliferation assay and differentiation assay

Cell proliferation was determined with a ^3^H-thymidine incorporation assay (Perkin Elmer, Waltham, USA). MSCs were plated in a 96 wells-plate at a concentration of 1000 cells per well. 25 µL of ^3^H-thymidine (5 mCi (185 MBq)) was added to the wells at 0, 24, 48 and 96 hours after plating. Time-point 0 hours (T0) was designated as 4 hours after plating, allowing the MSCs to attach to the plate. Incorporated ^3^H-thymidine was measured 16 hours later with a micro beta-plate counter (Perkin Elmer).

For the (non-contact) transwell co-culture differentiation assay [26], [27], 80.000 hMSCs were embedded in 0.2% HydroMatrix gel (Sigma-Aldrich) and cultured in transwell inserts for 48 hours (Millipore, Amsterdam, The Netherlands) in alpha-MEM with human platelet lysate supplement. 24 wells-plates were coated with 10 µg/mL Poly-L-Ornithine and 5 µg/mL Laminin (both Sigma-Aldrich) before plating mouse cortical neural stem cells (mNSC) (R&D systems, Minneapolis, USA) at a concentration of 25.000 cells per well in DMEM:F12 + B27 medium (Life Technologies). 20 ng/mL of human recombinant EGF (Peprotech, Rocky Hill, NJ, USA) and 20 ng/mL of mouse b-FGF (Peprotech) was added to the mNSCs cultures daily for the following 2 days. 48 h after plating the mNSCs, co-culture was started by transferring the inserts containing hMSCs (in HydroMatrix gel) into the 24-wells plates with mNSCs. The assay was stopped by fixing the mNSCs with 4% paraformaldehyde (PFA) at T0 (unstimulated mNSCs) and at T96 after starting co-culture with hMSCs. As a control mNSCs were co-cultured with inserts containing only 0.2% HydroMatrix gel and no hMSCs. Differentiation of the mNSCs was assessed by immunocytochemistry.

### Immunocytochemistry

Briefly, PFA-fixed mNSCs from the co-culture assay were blocked with 5% BSA and 0.1% saponin for 30 minutes followed by incubation for 1 hour at room temperature with primary antibodies: mouse anti-nestin (1∶200) (BD Biosciences, Breda, The Netherlands), rabbit anti-Olig2 (1∶400) (Millipore), mouse anti-GFAP (1∶100) (Acris antibodies, Herford, Germany) or rabbit-anti βIII-Tubulin (1∶1000) (Abcam antibodies, Cambridge, UK). Secondary antibodies goat anti-mouse AF488 or goat anti-rabbit AF594 (Invitrogen, Paisley, UK) were incubated for one hour at room temperature. Nuclei were counterstained with 4′6-diamidino-2-phenylindole (DAPI; Invitrogen) and mounted with FluoroSave reagent (Calbiochem, Nottingham, UK). Fluorescent images were taken with an AxioCam MRm (Carl Zeiss, Sliedrecht, The Netherlands) on an Axio Observer Microscope with Axiovision Rel 4.6 software (Carl Zeiss).

### MSC tracking

1×10^6^ hMSCs were labeled with PKH-26 Red fluorescent cell linker kit (Sigma-Aldrich) and administered intranasally to mouse pups at 10 days after induction of HI. 24 hours later, mice were perfused intracardially with PBS followed by 4% PFA. Fixed brains were cryoprotected in a sucrose gradient (15% followed by 30% overnight) and embedded in OCT compound (VWR BDH Prolab, Boxmeer, The Netherlands). Coronal cryosections (8 µm) were stained with DAPI (Invitrogen) for nuclei counterstaining. Fluorescent images were captured using an EMCCD camera (Leica Microsystems, Benelux) and Softworx software (Applied Precision, Washington, USA).

### Gene expression profiling

Real-time PCR analysis was done on pooled samples from 10 HI mouse pups and 6 sham-operated mouse pups on the RT^2^ Profiler PCR array (PAMM-022, SABiosciences, Venlo, Netherlands). A standard brain region was isolated at 10 and 17 days following HI induction or sham-operation by dissecting the ipsilateral hemisphere at bregma and 2 mm from bregma on ice and pulverizing on liquid nitrogen. Total RNA was isolated by TRIzol according to the manufacturer's instructions (Invitrogen). The amount of RNA was measured by spectrophotometry at 260 nm. The RNA quality was determined with the OD 260/280 ratio, which was between 1,9 and 2,1. To confirm that there was no RNA degradation, all samples were run on a 1% agarose gel. cDNA was synthetized with SuperScript Reverse Transcriptase (Invitrogen). Expression of 84 genes was measured according to the producer's guidelines by using the RT^2^ Real-Time SYBR green PCR master Mix (SABiosciences) on the Bio-rad IQ5 (Thermo Scientific, Waltham, MA, USA). Data was normalized for the expression of GAPDH and actin. Analysis was done with the PCR Array Data Analysis Software (SABiosciences). Two separate comparisons were made, i.e. the sham-operated group was compared to the 10 days after HI group and the 10 days after HI group was compared to the 17 days after HI group. Changes in gene expression were determined as significant by an arbitrary cut-off of >2-fold. The PCR array results were validated by quantitative reverse transcription (qRT)-PCR analysis on individual samples and pooled samples (Supplemental table 5).

### MSCs co-culture with brain extracts

10 days after HI-surgery, mice were euthanized by pentobarbital overdose, decapitated and brains were removed. The ipsilateral hemisphere was dissected on ice at 0 mm to 2 mm from bregma and was subsequently pulverized on liquid nitrogen. Dissected brains were dissolved in KO-DMEM medium (Gibco Life Technologies) at a final concentration of 150 mg/mL and centrifuged for 10 minutes at 3000 g at 4°C. Supernatants were collected as ‘brain extract’ and protein concentration was measured using a protein assay with BSA as a standard on a Multiskan GO (Thermo Scientific). hMSCs were cultured at a concentration of 40.000 cells per well in a 24 wells-plate for 24 hours before replacing the medium with knock-out medium with either 1 mg/mL HI brain extract or without extract. After 72 hours of culture with brain extracts, hMSCs were lysed for RNA isolation.

### RNA isolation and qPCR

Total RNA was isolated with the RNAmini kit according to the manufacturer's instructions (Invitrogen). The amount of RNA was measured with the nanodrop 2000 (Thermo Scientific). RNA quality was determined with the OD 260/280 ratio, which was between 1,9 and 2,1. cDNA was synthetized with SuperScript Reverse Transcriptase (Invitrogen). The expression of CXCR3 gene (Supplemental table 5) was measured by quantitative reverse transcription (qRT)-PCR (Biorad IQ5) analysis on individual samples. Data was normalized for the expression of GAPDH and β-actin.

### Sensorimotor function

Unilateral sensorimotor impairments were measured in the cylinder rearing test (CRT). Mice were placed in a transparent cylinder and the weight-bearing paw (left (impaired), right (unimpaired) or both) contacting the cylinder wall during full rear was scored. Paw preference was calculated as ((right - left)/(right + left + both)) ×100%.

### Histology

Coronal paraffin sections (8 µm) of paraformaldehyde (PFA)-fixed brains were incubated with mouse-anti-myelin basic protein (MBP) (Sternberger Monoclonals, Lutherville, MD,) or mouse-anti-microtubule-associated protein 2 (MAP2) (Sigma-Aldrich) followed by biotinylated horse-anti-mouse antibody (Vector Laboratories, Burliname, CA). Binding was visualized with Vectastain ABC kit (Vector Laboratories) and diaminobenzamidine. Ipsilateral MAP2 and MBP area loss was determined on sections corresponding to −1.85 mm from bregma in adult mouse brain. MBP and MAP2 staining were quantified using ImageJ software (http://rsb.info.nih.gov/ij/) and Adobe Photoshop CS5, respectively.

### Immunofluorescence

Coronal paraffin sections (8 µm) were blocked with 2% BSA and 0.1% saponin, incubated for 2 hours with primary antibodies rabbit anti-Iba1 (1∶200) (Wako Chemicals, Osaka, Japan) and mouse anti-GFAP (1∶100) (Acris antibodies) followed by incubation with secondary antibodies goat anti-rabbit AF594 and goat anti-mouse AF488 (both Invitrogen). Nuclei were counterstained with DAPI (Invitrogen) and mounted with FluoroSave reagent (Calbiochem). Fluorescent images were captured with an AxioCam MRm (Carl Zeiss, Sliedrecht, The Netherlands) on an Axio Observer Microscope with Axiovision Rel 4.6 (Carl Zeiss).

### Statistical analysis

Quantification of the differentiation assay, and GFAP and Iba-1 staining was done by measuring pixel intensity with ImageJ software (http://rsb.info.nih.gov/ij/). Analysis was performed blind to treatment groups to avoid bias. Similar thresholds were used for all treatment groups. The mean of all signal intensities from all individual pictures per sample were combined and this number was entered in the graph. For the GFAP and Iba-1 staining, we quantified one section per animal and 10 regions of interest with a 20× magnification. 7 cortical regions (3 fields in the motor cortex and 4 fields in the somatosensory cortex) were quantified. Of these 7 regions: 3 regions were just adjacent to the lesion border, in sham operated animals just above the corpus callosum (Cortical layer 6), 2 in the upper layers of the motor cortex and 2 in Layer 4 of the cortex. We also quantified 3 ROI's in the thalamic regions of the brain bordering the lesion cavity or the hippocampal structure in sham operated animals. We ensured consistency across animals by maintaining recognizable regions in the brain as landmarks. Examples of such landmarks are Layer 4 in the cortex and the corpus callosum for the vertical line and the size and structure of the hippocampus and corpus callosum for the horizontal line. Data are presented as mean and SEM. Statistical significance was determined by using one-way ANOVA followed by Bonferroni post-hoc tests. For the differentiation assay, statistical significance was assessed by Two-tailed unpaired T-test. p<0.05 was considered statistically significant.

## Results

### Pre-treatment characterization of human MSCs

**Figure 1:**
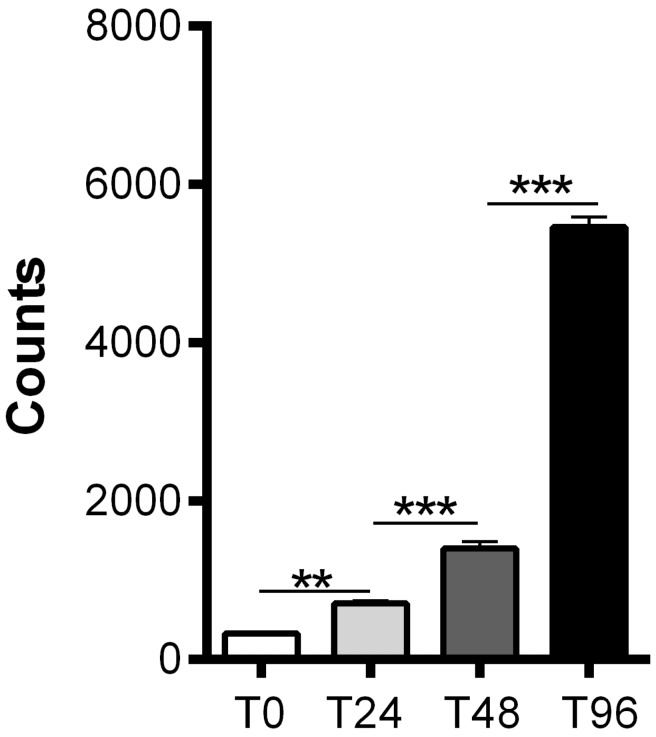
*In vitro* proliferation of hMSCs. Proliferating capacity of hMSCs *in vitro*. 1000 hMSCs were plated (T0) and proliferation was assessed at 4(T0), 24(T24), 48(T48) and 96(T96) hours after plating the MSCs by adding ^3^H-thymidine to the culture and measuring ^3^H-thymidine uptake 16 hours later. Data represent mean ± SEM. ** p<0.01; *** p<0.001 by ANOVA and Bonferroni post-hoc test. (n = 10 wells for each condition).

Before intranasal administration of hMSCs, we assessed expression of stem cell markers by the hMSCs. MSCs expressed CD73, CD90 and CD105 and contained less than 10% CD45 positive cells as confirmed by FACS analysis (Fig. S1). We also show that hMSCs are capable of proliferating *in vitro* (Fig. 1A and Table S1).

### Human MSCs induce mouse NSCs to differentiate *in vitro*

**Figure 2:**
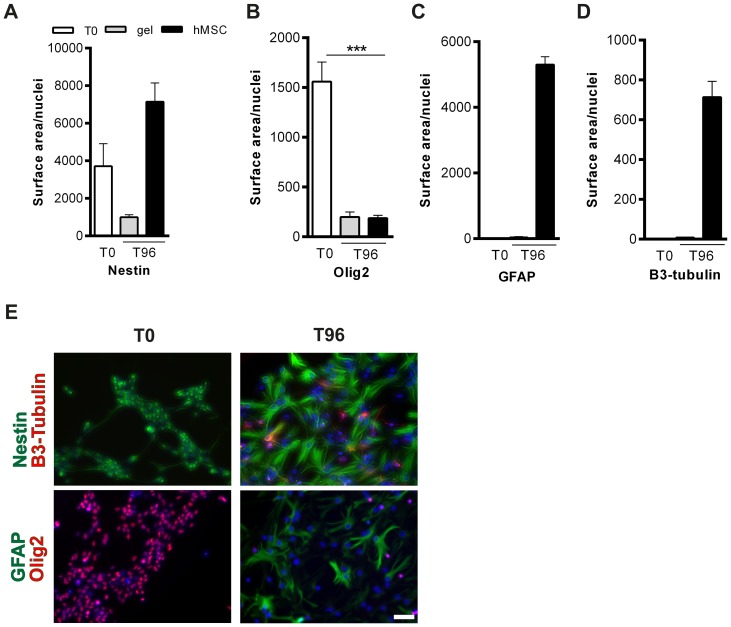
hMSCs induce differentiation of mouse NSCs *in vitro*. *In vitro* mNSC transwell differentiation assay in co-culture with hMSCs. mNSCs were fixed at 4(T0) and 96(T96) hours after co-culture with hMSCs and stained for (A) nestin (green), (B) Olig2 (red), (C) GFAP (green) and (D) βIII-Tubulin (red). Data represent mean ± SEM. *** p<0.001 by Unpaired two-tailed T-test. Scale bar  = 100 µm. (n = 4 wells per condition).

As the success of the *in vivo* experiment depends on the capacity of hMSCs to interact with the mouse host environment despite differences in species, we tested whether human MSCs were functional with respect to being capable of inducing expression of βIII-Tubulin by murine NSCs. βIII-Tubulin is a microtubule protein that is almost exclusively expressed by immature neurons [28]. To this end, we cultured mNSCs *in vitro* with hMSCs in a non-contact transwell assay and measured the effect of hMSCs on βIII-Tubulin and GFAP expression by NSCs. hMSCs were placed in a transwell insert on top of adherent mNSCs in a 24 wells-plate and co-cultured for 96 hours. Markers for nestin, Olig2, GFAP and βIII-Tubulin were used for cell fate determination. Our results show that the number of nestin^+^ cells, as a marker for undifferentiated stem cells, increased after co-culture with hMSCs (Fig. 2A and E; Table S2). Expression of the oligodendrocyte-progenitor marker Olig2 decreased after co-culture with hMSCs (Fig. 2B and E). Figure 2C shows that hMSCs were capable of inducing mNSC differentiation towards neuronal (i.e. βIII-Tubulin positive) cell fate (Fig. 2C and E). Moreover, our data show that hMSCs also induce mNSC differentiation into GFAP^+^/astrocytic cell fate (Fig. 2D and E). Without co-culture with hMSCs, mNSCs do not differentiate as they remain positive for the markers Nestin and Olig2 (Fig. 2A–D).

### hMSCs migrate towards the HI-induced cerebral lesion site

**Figure 3:**
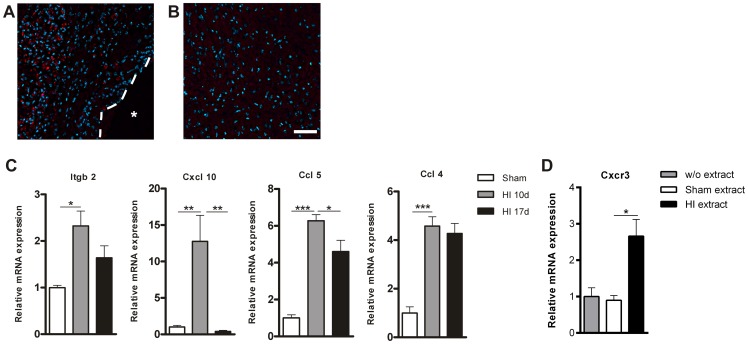
PKH-26 labeled hMSCs migrate to the lesion site. 1×10^6^ hMSCs were labeled with PKH-26 and administered intranasally at 10 days after HI. (A+B) Mice were terminated 24 hours after hMSC treatment. (A) PKH-26^+^ hMSCs in the ipsilateral damaged cortex. (B) Contralateral cortex shows no PKH-26^+^ signal. (C) qPCR validation of PCR array confirmed the upregulation of five genes 10 days after HI. At 17 days after HI 2 genes were down-regulated in comparison to 10 days following HI. (HI n = 10; sham n = 6). (D) hMSCs express CXCR3, which increases after co-culture with HI brain extract (n = 2). Data represent mean ± S.E.M. *p<0.05; **p<0.01; ***p<0.001 by ANOVA and Bonferroni post-hoc test. Dashed line  =  lesion border; Asterisk  =  lesion site. Blue  =  Dapi staining. Scale bar  = 50 µm.

In a previous study we showed that PKH-26-labeled C57BL/6 MSCs migrate specifically towards the HI-damaged brain region within 24 hours after intranasal administration [11]. We demonstrated that the PKH-26^+^ signal is specific and not due to auto-fluorescence of damaged tissue as we did not see any positive signal in the HI-damaged brain after vehicle-treatment. As we are applying human MSCs to the mouse brain, we first established if intranasal delivery is an efficient administration route before investigating the therapeutic potential of hMSCs *in vivo*. We administered 1×10^6^ PKH-26-labeled hMSCs intranasally at 10 days after HI and sacrificed the mice 24 hours later. At 10 days after HI, i.e. the time-point when hMSCs are administered intranasally, the entire hippocampus and part of the cortex had degenerated and an evident cyst could be discerned in the ipsilateral hemisphere. 24 hours after hMSC administration we observed a strong PKH-26^+^ signal, i.e. hMSCs, in the sensorimotor and epithalamic regions of the damaged brain, surrounding the cyst (Fig. 3A). We did not detect any PKH-26^+^ signal in the contralateral hemisphere (Fig. 3B).

We have previously shown that *mouse* MSCs no longer reach the lesion site when given at 17 days after HI [11]. Therefore, we investigated which chemotactic factors may be involved in regulating MSC migration towards the lesion, by comparing the expression profile of chemotactic factors at the lesion site from 17 days after HI with 10 days post-insult. First, we analyzed the chemokine profile induced by HI by comparing the gene expression profiles from HI mice to sham-operated mice at 10 days following the insult. To this end a 2 mm region from -2 bregma at the ipsilateral hemisphere was dissected and the expression of 84 chemotactic factors was analyzed by PCR array using an arbitrary cut off of >2.00 fold change (Table S3). We validated the results from the PCR array by qPCR, which confirmed that the factors Ccl4, Ccl5, Cxcl10 and Itgb-2 were significantly upregulated at 10 days following HI (Fig. 3C). Next we compared the gene expression profile of 84 chemotactic factors at the lesion site at 10 and 17 days following HI (Table S4 and S5). Validation of the PCR array results on individual samples by qPCR analysis confirmed that the expression of the chemokines Ccl5 and Cxcl10 were significantly down-regulated at 17 days in comparison to 10 days after HI. The expression of Ccl4 and Itgb-2 did not change significantly at 17 days (Fig. 3C).

To determine whether CXCL10 may be involved in mediating the migration of hMSCs towards the lesion, we performed a qPCR to assess whether hMSCs *in vitro* express CXCR3, the receptor for CXCL10. Our qPCR results show that hMSCs express CXCR3, without co-culturing the cells with HI brain extract. Interestingly, following 72 hours of co-culture with brain extracts from 10 days after HI we observed an increase in CXCR3 mRNA expression (Fig. 3D and Table S6).

### Intranasal hMSC treatment improves sensorimotor outcome and lesion volume after HI

**Figure 4:**
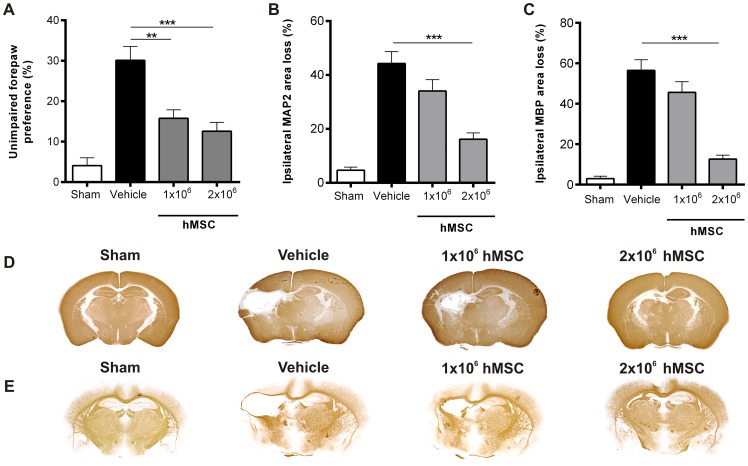
Dose effect of hMSC on motor performance and lesion volume. Mice were treated intranasally with either 1×10^6^ or 2×10^6^ hMSCs or vehicle at 10 days after HI. (A) Preference to use the unimpaired forepaw in the cylinder rearing test (CRT) was assessed at 28 days after HI. Sham-operated littermates (Sham) were used as controls. (B–C) Quantification of ipsilateral MAP2 (B) and MBP (C) area loss measured as 1- (ipsi-/contralateral MAP2- or MBP-positive area) at 28 days after HI. Representative sections of MAP2 (D) and MBP (E) staining. Data represent mean ± SEM. **p<0.01; ***p<0.001 by ANOVA and Bonferroni post-hoc test. Sham n = 13; Vehicle n = 21; 1×10^6^ hMSC n = 11; 2×10^6^ hMSC n = 12. Data presented in this figure are results from pups pooled out of 11 different litters. Treatment groups were randomly distributed between litters.

Next, we investigated whether hMSCs were also capable of improving sensorimotor behavior and decreasing lesion size in HI mice *in vivo*. To assess motor function we used the cylinder rearing test that measures the preference to use the unimpaired forepaw. We treated mouse pups at 10 days after HI with 1×10^6^ or 2×10^6^ hMSC or vehicle-treatment. Our results show that both doses of hMSCs significantly improved sensorimotor function at 21 (data not shown) and 28 days after HI as measured in the cylinder rearing test (Fig. 4A and Table S7).

Next we analyzed loss of MAP2 and MBP staining as measures for gray and white matter damage, respectively. Treatment with 2×10^6^ hMSCs substantially decreased MAP2 loss (Fig. 4B and D) and MBP loss (Fig. 4C and E) at 28 days after HI. Treatment with the lower dose of 1×10^6^ hMSCs was not sufficient to significantly reduce either gray (Fig. 4B and D; Table S8) or white matter injury (Fig. 4C and E; Table S9).

### GFAP and Iba-1 expression following hMSC treatment

**Figure 5:**
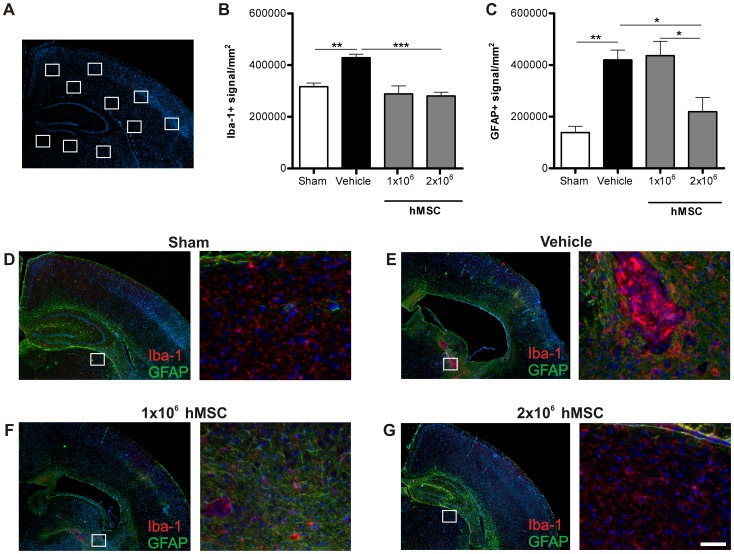
hMSCs reduce the activation of glial cells at 28 days after HI. Mice were treated with either 1×10^6^ or 2×10^6^ hMSCs or vehicle intranasally at 10 days following HI. Mice were sacrificed 28 days after HI. (A) Schematic overview of fields quantified. (B) Quantification of Iba-1+ signal/mm^2^ or (C) GFAP+ signal/mm^2^. (D–G) Representative sections of Iba-1 (red) and GFAP (green) expression after sham-operation (D), vehicle (E), 1×10^6^ hMSCs (F) or 2×10^6^ hMSCs (G). Sections are counterstained with DAPI (blue). Scale bar  = 100 µm. Data represent mean ± SEM. * p<0.05; **p<0.01; ***p<0.001 by ANOVA and Bonferroni post-hoc test (Sham and Vehicle n = 4; 1×10^6^ and 2×10^6^ MSC n = 3).

To assess whether treatment with hMSCs reduced scar formation in the long-term, we stained brain sections at 28 days after HI for astrocytes and microglia with the markers GFAP and Iba-1, respectively. We analyzed 10 regions in the brain as depicted in figure 5A. Following HI, a long-lasting upregulation of GFAP^+^ and Iba-1^+^ signal in the region adjacent to the cystic lesion can be discerned (Fig. 5B, C, E). Treatment with either 1 or 2×10^6^ hMSCs decreased Iba-1 expression to sham level (Fig. 5B, F, G and ale S10). Furthermore, the highest dose of 2×10^6^ hMSCs also significantly reduced GFAP expression to sham level (Fig. 5B, C, G), whereas the lower dose of 1×10^6^ hMSCs had no effect on GFAP expression (Fig. 5C and F; Table S11).

## Discussion

Our study shows that *human* MSCs have the capacity to promote neuroregeneration. This finding is reflected by our results showing that intranasal administration of hMSCs significantly improves motor behavior, and decreases lesion size and scar formation at 28 days after HI brain damage in neonatal mice. Furthermore, our *in vitro* results demonstrate that *human* MSCs are capable of inducing mNCSs to differentiate towards astrocytic and neuronal cell fate. This suggests that hMSCs do not need cell to cell contact with neural stem cells, but rather promote endogenous neurogenesis and lesion repair by the secretion of neurotrophic factors. We also show that hMSCs reach the damaged brain region in the mouse within 24 hours after intranasal administration. Importantly, our work also provides new insight into the chemotactic factors that may regulate MSC migration towards the lesion site. Our results show that the chemokine CXCL10 is strongly upregulated at 10 days following HI.

We have previously shown that intranasal treatment with both 0.5×10^6^ and 1×10^6^ murine MSCs decreased HI lesion volume substantially by 53% and 71%, respectively [11] and improved motor behavior by 47%. In the present work we demonstrate that hMSCs decrease gray and white matter lesion volume with respectively 63% and 78% and improve motor behavior by 58%. Our present work indicates that, provided that the optimal dose is used, hMSCs can be as effective as mouse MSCs and have a remarkable effect on both motor behavior and cerebral lesion size after HI (Fig. 4). These positive effects may result from increased neurogenesis as we observed that hMSCs have the capacity to induce mNSCs to differentiate into neurons *in vitro*. We also observed that hMSCs repair white matter structures, since MBP expression increases significantly at 28 days after HI (i.e. 18 days after MSC treatment). Although, the results of our *in vitro* differentiation assay did not show differentiation into oligodendrocyte lineage, unpublished work from our group, shows that *in vitro* Olig2 expression increases when MSCs are genetically engineered to promote differentiation towards the oligodendrocyte lineage. These findings are in line with previous studies showing that neuronal cell differentiation requires downregulation of oligodendrocyte transcription factor 2 (Olig2), as this is a strong inhibitor of neurogenesis *in vitro* and *in vivo* [29], [30].

Importantly, we also showed in a recent paper that at 10 days following the insult the lesion is fully developed and does not progress further, which strongly supports a regenerative rather than neuroprotective effect of MSCs [31]. We also provide evidence that MSC induce lesion repair by boosting the endogenous neuroregenerative capacity. Our results show that intranasal treatment with mouse MSCs significantly increases GFAP/Nestin and DCX expression in the subventricular zone (SVZ) and lesion site (at the hippocampal level) 1–3 days after administration. Moreover, we observed a dramatic increase in the number of NeuN^+^ cells that repopulate the neocortex and hippocampal region at 5 days following MSC treatment. Cortex layer 4 can be clearly distinguished at 5 days and the different hippocampal regions can be clearly discerned at 18 days after MSC [31]. Also in a previous paper we showed that intracranial MSC administration increases the number of BrdU^+^/NeuN^+^ cells and BrdU^+^/Olig2^+^ cells in the hippocampus and cortex 18 days after administration [13]. Moreover, we did not find any evidence of MSC engraftment in the brain parenchyma, which indicates that the neurogenesis observed is host-derived. In addition, extensive histo-pathological studies demonstrated that MSC administration does not induce malignancies or other lesions as measured 14 months after the insult (manuscript submitted). Instead, we found that at 72 hours after administration, the number of PKH26^+^-MSCs has decreased by more than 80% [31].

Interestingly, the lower dose of hMSCs, which did not have an effect on lesion volume, only decreased microglia activation and had no effect on astrocyte activation. Moreover, the lower dose of hMSC (1×10^6^) improved motor behavior, but did not decrease gray or white matter loss. In a previous study by Lee JA *et al*. [32], hMSCs were administered intracardially 3 days after HI induction in the neonatal rat without any positive effect on lesion size. However, in contrast to our study, the authors only tested one dose of hMSCs i.e. 1×10^6^, which also had no effect in our study. Furthermore, the intracardial administration route may be a less efficient delivery method than the intranasal route, as systemic delivery may result in a smaller number of hMSCs homing to the injured brain. There are other studies on stem cell administration following HI injury that describe restoration of behavior without significant decrease of lesion volume. One possible explanation is that downregulation of inflammation may restore motor neuron function and thus also motor behavior. In a previous study, we showed that although there is no gross neuronal loss in the motor cortex following HI, axonal connectivity is impaired. Indeed, following HI there is an increase in axonal rewiring to the unlesioned motor cortex [15]. Moreover, we have shown that MSC treatment restores cortical connectivity at 11 days following intracranial MSC administration using anterograde and retrograde labeling. At 18 days following intracranial MSC treatment there is also an increase in synaptophysin expression, a marker for synaptogenesis [13], [15].

We observed that vehicle-treated mice with extensive HI-induced cerebral cell loss also show substantial scar formation at 28 days after HI (i.e. 18 days after MSC treatment) (Fig. 5). In contrast, mice that received hMSC treatment showed decreased lesion size, which is associated with decreased astrocyte and microglia activation. In agreement with these results, we described in a recent paper that MSC treatment resolves the glial scar as Iba-1 and GFAP positive fluorescence significantly decreases over time, returning to sham level at 18 days following treatment [31]. Reactive astrocytes contribute to a process called glial scar formation, which forms a physical and chemical barrier that prevents inflammation from spreading through the tissue, thus restricting the progression of the injury. However, a downside of scar formation is that it also inhibits growth cone motility, thereby impairing axon regeneration [33]–[35]. This may be one of the reasons underlying impaired neurogenesis following a HI insult in the neonatal brain [36]. Hence, our data suggest that hMSC-induced reduction in astrocyte activation is crucial for repair of the lesion after HI brain damage. The decrease in astrocyte and microglia activity may be mediated by anti-inflammatory cytokines secreted by the hMSCs. We and others have previously shown that MSCs secrete IL-10 [9], [16], which is known to suppress the pro-inflammatory phenotype of both microglia and astrocytes [37]. Interestingly, the effect of MSCs on GFAP positive cells is apparently restricted to reactive astrocytes involved in astrogliosis, as GFAP expression can still be observed in the hippocampus following repair and the staining strongly resembles the hippocampus of sham-operated animals. Future studies should focus on the mechanisms underlying MSC-mediated reduction of astrocytic scar in brain lesions, which may be crucial in promoting a pro-neurogenic microenvironment that supports tissue repair.

Besides showing the potential of hMSCs to repair the HI injured brain, we also provide new insight into factors that may be involved in MSC migration from the nose to the injury site. Our results show that expression of the integrin beta 2 protein (Itgb-2) is upregulated at 10 days after HI. Itgb-2 together with the Intercellular Adhesion Molecule 1 (ICAM-1) mediates the migration of leukocytes along endothelial cells [38], [39] and may be involved in the migration of MSCs through blood vessels and regulate transmigration into the brain tissue. The results from the PCR array show that CXCL10 is the chemotactic factor with the highest fold change at 10 days following HI. Interestingly, at 17 days after HI, the expression level of this chemokine has returned to sham level (Fig. 3C). We also show that the expression of the CXCL10 receptor, CXCR3, increases following co-culture of hMSCs with brain extract from 10 days after HI (Fig. 3D). Together these results suggest that CXCL10 may play an important role in regulating homing of MSCs to the lesion site. We also found that Ccl5 significantly decreases at 17 days following HI, which also suggests a role for this chemokine in MSC homing to lesion. Hence, our data propose that Cxcl10 and Ccl5 secreted by astrocytes, microglia and neurons [40] at the lesion site attract MSCs to home at the lesion site.

## Conclusions

To our knowledge this is the first study that shows the potent regenerative effects of intranasally administered *human* MSCs on HI brain damage in the neonatal mouse. Moreover, our results suggest that the decrease in glial scar formation induced by hMSCs is a crucial step in promoting neurogenesis. Finally, the efficiency of the intranasal delivery route was confirmed, as hMSCs migrate specifically towards the lesion site in the mouse brain. The results in this study strongly support the therapeutic potential of hMSCs for neonatal HI.

## Supporting Information

Figure S1 **Characterization of hMSCs by FACS analysis.** (A) isotype control and (B) antigen expression (CD90, CD105, CD73 and CD45).
(TIF)

Table S1 **Raw data of measurements shown in “** **Figure 1** **.** ***In vitro*** **proliferation of hMSCs”.**
(DOCX)

Table S2 **Raw data of measurements shown in “** **Figure 2** **. hMSCs induce differentiation of mouse NSCs** ***in vitro*** **”.**
(DOCX)

Table S3 **Down- or upregulated genes at 10 days after HI in comparison to sham-operated mice.**
(DOCX)

Table S4 **Down- or upregulated genes at 17 days after HI in comparison to 10 days after HI.**
(DOCX)

Table S5 **List of primers used for array validation on qRT-PCR.**
(DOCX)

Table S6 **Raw data of measurements shown in “** **Figure 3** **. PKH-26 labeled hMSCs migrate to the lesion site”.**
(DOCX)

Table S7 **Raw data of CRT measurements shown in “** **Figure 4** **. Dose effect of hMSC on motor performance and lesion volume”.** (Outlier detected with Grubbs test p<0.05).
(DOCX)

Table S8 **Raw data of MBP measurements shown in “** **Figure 4** **. Dose effect of hMSC on motor performance and lesion volume”.**
(DOCX)

Table S9 **Raw data of MAP2 measurements shown in “** **Figure 4** **. Dose effect of hMSC on motor performance and lesion volume”.**
(DOCX)

Table S10 **Raw data of Iba-1^+^ signal measurements shown in “** **Figure 5** **. hMSCs reduce the activation of glial cells at 28 days after HI”.**
(DOCX)

Table S11 **Raw data of GFAP^+^ signal measurements shown in “Figure 5. hMSCs reduce the activation of glial cells at 28 days after HI”.**
(DOCX)
