# Supplementary figures and images for: Intranasal Administration of Human MSC for Ischemic Brain Injury in the Mouse: In Vitro and In Vivo Neuroregenerative Functions
Source: PLoS One. 2014 Nov 14;9(11):e112339. doi: 10.1371/journal.pone.0112339 (PMC4232359; doi:10.1371/journal.pone.0112339)

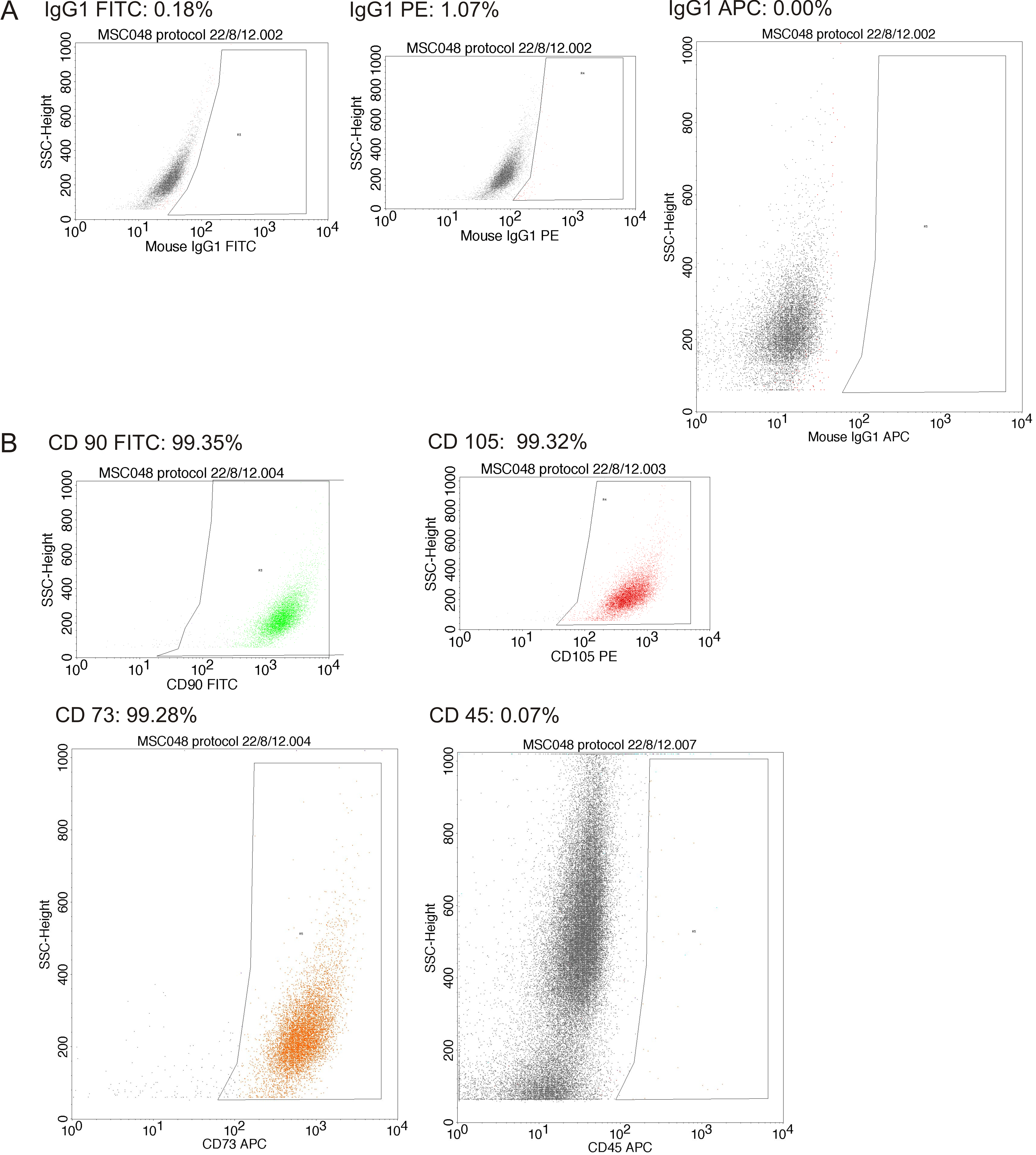

Supplement: Figure S1 — Characterization of hMSCs by FACS analysis. (A) isotype control and (B) antigen expression (CD90, CD105, CD73 and CD45). (TIF) [file pone.0112339.s001.tif]
