# Supplementary material for: Intranasal Administration of Human MSC for Ischemic Brain Injury in the Mouse: In Vitro and In Vivo Neuroregenerative Functions
Source: PLoS One. 2014 Nov 14;9(11):e112339. doi: 10.1371/journal.pone.0112339 (PMC4232359; doi:10.1371/journal.pone.0112339)
Supplement: Table S1 — Raw data of measurements shown in “ Figure 1 . In vitro proliferation of hMSCs”. (DOCX) [file pone.0112339.s002.docx]

**Table S1**

| T0  hMSC |  | T24  hMSC |  | T48  hMSC |  | T96  hMSC |
| --- | --- | --- | --- | --- | --- | --- |
| 372,54 |  | 886,54 |  | 897,05 |  | 6265,75 |
| 312,54 |  | 645,54 |  | 1141,05 |  | 5686,75 |
| 346,54 |  | 726,54 |  | 1522,05 |  | 5681,75 |
| 329,54 |  | 542,54 |  | 1761,05 |  | 4786,75 |
| 296,54 |  | 643,54 |  | 1728,05 |  | 5134,75 |
| 252,54 |  | 853,54 |  | 1407,05 |  | 5637,75 |
| 309,54 |  | 676,54 |  | 1351,05 |  | 5538,75 |
| 284,54 |  | 729,54 |  | 1169,05 |  | 5501,75 |
| 437,54 |  | 603,54 |  | 1588,05 |  | 5356,75 |
| 361,54 |  | 761,54 |  | 1471,05 |  | 5020,75 |
