# Supplementary material for: Intranasal Administration of Human MSC for Ischemic Brain Injury in the Mouse: In Vitro and In Vivo Neuroregenerative Functions
Source: PLoS One. 2014 Nov 14;9(11):e112339. doi: 10.1371/journal.pone.0112339 (PMC4232359; doi:10.1371/journal.pone.0112339)
Supplement: Table S2 — Raw data of measurements shown in “ Figure 2 . hMSCs induce differentiation of mouse NSCs in vitro ”. (DOCX) [file pone.0112339.s003.docx]

**Table S2**

| Olig2 | | |  | | Nestin | | |  | GFAP |  | βIII-Tubulin |
| --- | --- | --- | --- | --- | --- | --- | --- | --- | --- | --- | --- |
| T0 |  | **T96 hMSC** | |  | **T0** |  | **T96 hMSC** | | **T96 hMSC** | | **T96 hMSC** |
| 1217,606 |  | 333,304 | |  | 2752,096 |  | 7651,444 | | 5910,911 | | 130,489 |
| 1004,408 |  | 358,875 | |  | 1002,701 |  | 4148,432 | | 4658,100 | | 21,705 |
| 922,642 |  | 138,059 | |  | 2432,383 |  | 5765,155 | | 4335,706 | | 357,446 |
| 1542,091 |  | 84,679 | |  | 0,000 |  | 8200,640 | | 4670,048 | | 571,320 |
| 1461,527 |  | 267,287 | |  | 2553,306 |  | 5152,363 | | 5499,904 | | 1411,418 |
| 1248,232 |  | 171,597 | |  | 2330,200 |  | 6054,909 | | 5832,661 | | 654,626 |
| 1036,390 |  | 126,646 | |  | 2815,359 |  | 2804,684 | | 6741,570 | | 676,079 |
| 1272,527 |  | 103,571 | |  | 6318,000 |  | 9544,570 | | 5950,790 | | 358,269 |
| 1646,869 |  | 210,631 | |  | 3649,507 |  | 10588,940 | | 5619,959 | | 367,177 |
| 2717,852 |  | 60,721 | |  | 1898,641 |  | 4461,978 | | 4890,047 | | 426,000 |
| 1920,363 |  | 198,985 | |  | 1523,264 |  | 14201,280 | | 4228,912 | | 910,469 |
| 54,431 |  |  | |  | 3029,063 |  |  | |  | | 1309,324 |
| 1062,724 |  |  | |  | 3501,260 |  |  | |  | | 1545,245 |
| 1406,121 |  |  | |  | 691,409 |  |  | |  | | 854,400 |
| 4208,772 |  |  | |  | 666,046 |  |  | |  | | 1000,754 |
| 859,810 |  |  | |  | 2376,544 |  |  | |  | | 951,095 |
| 1575,216 |  |  | |  | 7795,216 |  |  | |  | | 1030,037 |
| 1548,087 |  |  | |  | 2936,909 |  |  | |  | | 734,128 |
| 1450,812 |  |  | |  | 1696,192 |  |  | |  | | 496,527 |
| 3501,649 |  |  | |  | 3008,485 |  |  | |  | | 606,103 |
| 599,710 |  |  | |  | 1086,521 |  |  | |  | | 433,444 |
| 2043,130 |  |  | |  | 27666,500 |  |  | |  | | 1066,920 |
|  |  |  | |  |  |  |  | |  | | 681,200 |
|  |  |  | |  |  |  |  | |  | | 535,862 |
