# Supplementary material for: Intranasal Administration of Human MSC for Ischemic Brain Injury in the Mouse: In Vitro and In Vivo Neuroregenerative Functions
Source: PLoS One. 2014 Nov 14;9(11):e112339. doi: 10.1371/journal.pone.0112339 (PMC4232359; doi:10.1371/journal.pone.0112339)
Supplement: Table S3 — Down- or upregulated genes at 10 days after HI in comparison to sham-operated mice. (DOCX) [file pone.0112339.s004.docx]

| RefSeq | | Symbol | Description | Fold Up- or  Down-  regulation |
| --- | --- | --- | --- | --- |
| NM_001173550.1 | | C5ar1 | Complement component 5a receptor 1 | **-3,39** |
| NM_021609.3 | | Ccbp2 | Chemokine binding protein 2 | -1,19 |
| [NM_011329.3](http://www.ncbi.nlm.nih.gov/nuccore/NM_011329.3) | | Ccl1 | Chemokine (C-C motif) ligand 1 | -1,24 |
| [NM_011330.3](http://www.ncbi.nlm.nih.gov/nuccore/NM_011330.3) | | Ccl11 | Chemokine (C-C motif) ligand 11 | 1,44 |
| [NM_011331.2](http://www.ncbi.nlm.nih.gov/nuccore/NM_011331.2) | | Ccl12 | Chemokine (C-C motif) ligand 12 | **3,52** |
| [NM_011332.3](http://www.ncbi.nlm.nih.gov/nuccore/NM_011332.3) | | Ccl17 | Chemokine (C-C motif) ligand 17 | 1,15 |
| [NM_011888.2](http://www.ncbi.nlm.nih.gov/nuccore/NM_011888.2) | | Ccl19 | Chemokine (C-C motif) ligand 19 | 1,35 |
| [NM_011333.3](http://www.ncbi.nlm.nih.gov/nuccore/NM_011333.3) | | Ccl2 | Chemokine (C-C motif) ligand 2 | **7,66** |
| [NM_001159738.1](http://www.ncbi.nlm.nih.gov/nuccore/NM_001159738.1) | | Ccl20 | Chemokine (C-C motif) ligand 20 | 1,87 |
| [NM_009137.2](http://www.ncbi.nlm.nih.gov/nuccore/NM_009137.2) | | Ccl22 | Chemokine (C-C motif) ligand 22 | 1,22 |
| [NM_019577.4](http://www.ncbi.nlm.nih.gov/nuccore/NM_019577.4) | | Ccl24 | Chemokine (C-C motif) ligand 24 | -1,34 |
| [NM_009138.3](http://www.ncbi.nlm.nih.gov/nuccore/NM_009138.3) | | Ccl25 | Chemokine (C-C motif) ligand 25 | -1,34 |
| [NM_001013412.2](http://www.ncbi.nlm.nih.gov/nuccore/NM_001013412.2) | | Ccl26 | Chemokine (C-C motif) ligand 26 | -1,99 |
| [NM_020279.3](http://www.ncbi.nlm.nih.gov/nuccore/NM_020279.3) | | Ccl28 | Chemokine (C-C motif) ligand 28 | 1,08 |
| [NM_011337.2](http://www.ncbi.nlm.nih.gov/nuccore/NM_011337.2) | | Ccl3 | Chemokine (C-C motif) ligand 3 | **3,78** |
| [NM_013652.2](http://www.ncbi.nlm.nih.gov/nuccore/NM_013652.2) | | Ccl4 | Chemokine (C-C motif) ligand 4 | **3,40** |
| [NM_013653.3](http://www.ncbi.nlm.nih.gov/nuccore/NM_013653.3) | | Ccl5 | Chemokine (C-C motif) ligand 5 | **4,98** |
| [NM_009139.3](http://www.ncbi.nlm.nih.gov/nuccore/NM_009139.3) | | Ccl6 | Chemokine (C-C motif) ligand 6 | **2,09** |
| [NM_013654.3](http://www.ncbi.nlm.nih.gov/nuccore/NM_013654.3) | | Ccl7 | Chemokine (C-C motif) ligand 7 | **4,37** |
| [NM_021443.3](http://www.ncbi.nlm.nih.gov/nuccore/NM_021443.3) | | Ccl8 | Chemokine (C-C motif) ligand 8 | 1,81 |
| [NM_011338.2](http://www.ncbi.nlm.nih.gov/nuccore/NM_011338.2) | | Ccl9 | Chemokine (C-C motif) ligand 9 | 1,17 |
| [NM_009912.4](http://www.ncbi.nlm.nih.gov/nuccore/NM_009912.4) | | Ccr1 | Chemokine (C-C motif) receptor 1 | 1,21 |
| [NM_007721.4](http://www.ncbi.nlm.nih.gov/nuccore/NM_007721.4) | | Ccr10 | Chemokine (C-C motif) receptor 10 | -1,14 |
| [NM_007718.3](http://www.ncbi.nlm.nih.gov/nuccore/NM_007718.3) | | Ccr1l1 | Chemokine (C-C motif) receptor 1-like 1 | 1,08 |
| [NM_009915.2](http://www.ncbi.nlm.nih.gov/nuccore/NM_009915.2) | | Ccr2 | Chemokine (C-C motif) receptor 2 | **3,02** |
| RefSeq | **Symbol** | | **Description** | **Fold Up- or**  **Down-**  **regulation** |
| [NM_009914.4](http://www.ncbi.nlm.nih.gov/nuccore/NM_009914.4) | | Ccr3 | Chemokine (C-C motif) receptor 3 | 1,36 |
| [NM_009916.2](http://www.ncbi.nlm.nih.gov/nuccore/NM_009916.2) | | Ccr4 | Chemokine (C-C motif) receptor 4 | **-4,15** |
| [NM_009917.5](http://www.ncbi.nlm.nih.gov/nuccore/NM_009917.5) | | Ccr5 | Chemokine (C-C motif) receptor 5 | 1,31 |
| [NM_001190333.1](http://www.ncbi.nlm.nih.gov/nuccore/NM_001190333.1) | | Ccr6 | Chemokine (C-C motif) receptor 6 | -1,56 |
| [NM_007719.2](http://www.ncbi.nlm.nih.gov/nuccore/NM_007719.2) | | Ccr7 | Chemokine (C-C motif) receptor 7 | 1,49 |
| [NM_007720.2](http://www.ncbi.nlm.nih.gov/nuccore/NM_007720.2) | | Ccr8 | Chemokine (C-C motif) receptor 8 | -1,32 |
| [NM_001166625.1](http://www.ncbi.nlm.nih.gov/nuccore/NM_001166625.1) | | Ccr9 | Chemokine (C-C motif) receptor 9 | 1,19 |
| [NM_145700.2](http://www.ncbi.nlm.nih.gov/nuccore/NM_145700.2) | | Ccrl1 | Chemokine (C-C motif) receptor-like 1 | 1,17 |
| [NM_017466.4](http://www.ncbi.nlm.nih.gov/nuccore/NM_017466.4) | | Ccrl2 | Chemokine (C-C motif) receptor-like 2 | 1,28 |
| [NM_008153.3](http://www.ncbi.nlm.nih.gov/nuccore/NM_008153.3) | | Cmklr1 | Chemokine-like receptor 1 | 1,29 |
| [NM_027022.4](http://www.ncbi.nlm.nih.gov/nuccore/NM_027022.4) | | Cmtm2a | CKLF-like MARVEL transmembrane domain containing 2A | 1,54 |
| [NM_024217.3](http://www.ncbi.nlm.nih.gov/nuccore/NM_024217.3) | | Cmtm3 | CKLF-like MARVEL transmembrane domain containing 3 | 1,20 |
| [NM_153582.5](http://www.ncbi.nlm.nih.gov/nuccore/NM_153582.5) | | Cmtm4 | CKLF-like MARVEL transmembrane domain containing 4 | -1,04 |
| [NM_026066.2](http://www.ncbi.nlm.nih.gov/nuccore/NM_026066.2) | | Cmtm5 | CKLF-like MARVEL transmembrane domain containing 5 | 1,03 |
| [NM_026036.3](http://www.ncbi.nlm.nih.gov/nuccore/NM_026036.3) | | Cmtm6 | CKLF-like MARVEL transmembrane domain containing 6 | 1,11 |
| [NM_009142.3](http://www.ncbi.nlm.nih.gov/nuccore/NM_009142.3) | | Cx3cl1 | Chemokine (C-X3-C motif) ligand 1 | -1,06 |
| [NM_009987.4](http://www.ncbi.nlm.nih.gov/nuccore/NM_009987.4) | | Cx3cr1 | Chemokine (C-X3-C) receptor 1 | 1,19 |
| [NM_008176.3](http://www.ncbi.nlm.nih.gov/nuccore/NM_008176.3) | | Cxcl1 | Chemokine (C-X-C motif) ligand 1 | -1,34 |
| [NM_021274.2](http://www.ncbi.nlm.nih.gov/nuccore/NM_021274.2) | | Cxcl10 | Chemokine (C-X-C motif) ligand 10 | **12,79** |
| [NM_019494.1](http://www.ncbi.nlm.nih.gov/nuccore/NM_019494.1) | | Cxcl11 | Chemokine (C-X-C motif) ligand 11 | 1,91 |
| [NM_001012477.2](http://www.ncbi.nlm.nih.gov/nuccore/NM_001012477.2) | | Cxcl12 | Chemokine (C-X-C motif) ligand 12 | -1,00 |
| [NM_018866.2](http://www.ncbi.nlm.nih.gov/nuccore/NM_018866.2) | | Cxcl13 | Chemokine (C-X-C motif) ligand 13 | **2,42** |
| [NM_019568.2](http://www.ncbi.nlm.nih.gov/nuccore/NM_019568.2) | | Cxcl14 | Chemokine (C-X-C motif) ligand 14 | 1,34 |
| RefSeq | | **Symbol** | **Description** | **Fold Up- or**  **Down-**  **regulation** |
| [NM_011339.2](http://www.ncbi.nlm.nih.gov/nuccore/NM_011339.2) | | Cxcl15 | Chemokine (C-X-C motif) ligand 15 | -1,34 |
| [NM_023158.6](http://www.ncbi.nlm.nih.gov/nuccore/NM_023158.6) | | Cxcl16 | Chemokine (C-X-C motif) ligand 16 | **2,09** |
| [NM_009140.2](http://www.ncbi.nlm.nih.gov/nuccore/NM_009140.2) | | Cxcl2 | Chemokine (C-X-C motif) ligand 2 | 1,79 |
| [NM_203320.2](http://www.ncbi.nlm.nih.gov/nuccore/NM_203320.2) | | Cxcl3 | Chemokine (C-X-C motif) ligand 3 | 1,17 |
| [NM_009141.2](http://www.ncbi.nlm.nih.gov/nuccore/NM_009141.2) | | Cxcl5 | Chemokine (C-X-C motif) ligand 5 | **3,02** |
| [NM_008599.4](http://www.ncbi.nlm.nih.gov/nuccore/NM_008599.4) | | Cxcl9 | Chemokine (C-X-C motif) ligand 9 | -1,19 |
| [NM_178241.4](http://www.ncbi.nlm.nih.gov/nuccore/NM_178241.4) | | Cxcr1 | Chemokine (C-X-C motif) receptor 1 | -1,29 |
| [NM_009909.3](http://www.ncbi.nlm.nih.gov/nuccore/NM_009909.3) | | Cxcr2 | Chemokine (C-X-C motif) receptor 2 | 1,22 |
| [NM_009910.2](http://www.ncbi.nlm.nih.gov/nuccore/NM_009910.2) | | Cxcr3 | Chemokine (C-X-C motif) receptor 3 | **2,90** |
| [NM_009911.3](http://www.ncbi.nlm.nih.gov/nuccore/NM_009911.3) | | Cxcr4 | Chemokine (C-X-C motif) receptor 4 | **2,12** |
| [NM_007551.2](http://www.ncbi.nlm.nih.gov/nuccore/NM_007551.2) | | Cxcr5 | Chemokine (C-X-C motif) receptor 5 | -1,59 |
| [NM_030712.4](http://www.ncbi.nlm.nih.gov/nuccore/NM_030712.4) | | Cxcr6 | Chemokine (C-X-C motif) receptor 6 | 1,09 |
| [NM_001271607.1](http://www.ncbi.nlm.nih.gov/nuccore/NM_001271607.1) | | Cxcr7 | Chemokine (C-X-C motif) receptor 7 | 1,03 |
| [NM_010045.2](http://www.ncbi.nlm.nih.gov/nuccore/NM_010045.2) | | Darc | Duffy blood group, chemokine receptor | 1,11 |
| [NM_013521.2](http://www.ncbi.nlm.nih.gov/nuccore/NM_013521.2) | | Fpr1 | Formyl peptide receptor 1 | -1,11 |
| [NM_001025381.2](http://www.ncbi.nlm.nih.gov/nuccore/NM_001025381.2) | | Gpr17 | G protein-coupled receptor 17 | -1,15 |
| [NM_010431.2](http://www.ncbi.nlm.nih.gov/nuccore/NM_010431.2) | | Hif1a | Hypoxia inducible factor 1, alpha subunit | 1,03 |
| [NM_008337.3](http://www.ncbi.nlm.nih.gov/nuccore/NM_008337.3) | | Ifng | Interferon gamma | 1,50 |
| [NM_010551.3](http://www.ncbi.nlm.nih.gov/nuccore/NM_010551.3) | | Il16 | Interleukin 16 | 1,15 |
| [NM_008361.3](http://www.ncbi.nlm.nih.gov/nuccore/NM_008361.3) | | Il1b | Interleukin 1 beta | **2,58** |
| [NM_021283.2](http://www.ncbi.nlm.nih.gov/nuccore/NM_021283.2) | | Il4 | Interleukin 4 | -1,37 |
| [NM_031168.1](http://www.ncbi.nlm.nih.gov/nuccore/NM_031168.1) | | Il6 | Interleukin 6 | 1,19 |
| [NM_001082960.1](http://www.ncbi.nlm.nih.gov/nuccore/NM_001082960.1) | | Itgam | Integrin, alpha M | 1,52 |
| [NM_008404.4](http://www.ncbi.nlm.nih.gov/nuccore/NM_008404.4) | | Itgb2 | Integrin beta 2 | **3,31** |
| RefSeq | **Symbol** | | **Description** | **Fold Up- or**  **Down-**  **regulation** |
| [NM_001038663.1](http://www.ncbi.nlm.nih.gov/nuccore/NM_001038663.1) | Mapk1 | | Mitogen-activated protein kinase 1 | -1,08 |
| [NM_001168508.1](http://www.ncbi.nlm.nih.gov/nuccore/NM_001168508.1) | | Mapk14 | Mitogen-activated protein kinase 14 | -1,18 |
| [NM_019932.4](http://www.ncbi.nlm.nih.gov/nuccore/NM_019932.4) | | Pf4 | Platelet factor 4 | -1,07 |
| [NM_023785.2](http://www.ncbi.nlm.nih.gov/nuccore/NM_023785.2) | | Ppbp | Pro-platelet basic protein | **-2,37** |
| [NM_178804.3](http://www.ncbi.nlm.nih.gov/nuccore/NM_178804.3) | | Slit2 | Slit homolog 2 (Drosophila) | 1,01 |
| [NM_011577.1](http://www.ncbi.nlm.nih.gov/nuccore/NM_011577.1) | | Tgfb1 | Transforming growth factor, beta1 | 1,53 |
| [NM_011905.3](http://www.ncbi.nlm.nih.gov/nuccore/NM_011905.3) | | Tlr2 | Toll-like receptor 2 | **2,96** |
| [NM_021297.2](http://www.ncbi.nlm.nih.gov/nuccore/NM_021297.2) | | Tlr4 | Toll-like receptor 4 | 1,91 |
| [NM_013693.2](http://www.ncbi.nlm.nih.gov/nuccore/NM_013693.2) | | Tnf | Tumor necrosis factor | **2,04** |
| [NM_138302.1](http://www.ncbi.nlm.nih.gov/nuccore/NM_138302.1) | | Tymp | Thymidine phosphorylase | -1,28 |
| [NM_008510.1](http://www.ncbi.nlm.nih.gov/nuccore/NM_008510.1) | | Xcl1 | Chemokine (C motif) ligand 1 | 1,05 |
| [NM_011798.4](http://www.ncbi.nlm.nih.gov/nuccore/NM_011798.4) | | Xcr1 | Chemokine (C motif) receptor 1 | -1,18 |

**Table S3**.
