# Supplementary material for: Intranasal Administration of Human MSC for Ischemic Brain Injury in the Mouse: In Vitro and In Vivo Neuroregenerative Functions
Source: PLoS One. 2014 Nov 14;9(11):e112339. doi: 10.1371/journal.pone.0112339 (PMC4232359; doi:10.1371/journal.pone.0112339)
Supplement: Table S5 — List of primers used for array validation on qRT-PCR. (DOCX) [file pone.0112339.s006.docx]

**Table S5**

| Symbol | RefSeq | Forward (5’ 🡪 3’ ) | Reverse (3’ 🡪 5’) |
| --- | --- | --- | --- |
| Ccl4 | NM_013652.2 | gccctctctctcctcttgct | ggagggtcagagcccatt |
| Ccl5 | NM_013653.3 | tgcagaggactctgagacagc | gagtggtgtccgagccata |
| Cxcl10 | NM_021274.1 | gctgccgtcattttctgc | tctcactggcccgtcatc |
| Itgb2 | NM_008404.4 | cccagtgtgagtgtcagtgc | tcccaatgtagccagactca |
| Murine GAPDH | NM_008084.3 | tgaagcaggcatctgaggg | cgaaggtggaagagtgggag |
| Murine β-actin | NM_007393.3 | agagggaaatcgtgcgtgac | caatagtgatgacctggccgt |
| hCxcr3 | NM_001142797.1 | tgcctttgtaggggtcaagt | ctcacaagcccgagtaggag |
| hGAPDH | NM_001256799.2 | gaaggtgaaggtcggagtc | gaagatggtgatgggatttc |
| hβ-actin | NM_001101.3 | cctggcacccagcacaat | gggccggactcgtcatact |
