# Supplementary material for: Intranasal Administration of Human MSC for Ischemic Brain Injury in the Mouse: In Vitro and In Vivo Neuroregenerative Functions
Source: PLoS One. 2014 Nov 14;9(11):e112339. doi: 10.1371/journal.pone.0112339 (PMC4232359; doi:10.1371/journal.pone.0112339)
Supplement: Table S6 — Raw data of measurements shown in “ Figure 3 . PKH-26 labeled hMSCs migrate to the lesion site”. (DOCX) [file pone.0112339.s007.docx]

**Tabel S6**

| Sham extract | HI extract | w/o extract |
| --- | --- | --- |
| 0,766593 | 2,201388 | 0,757264 |
| 1,028129 | 3,120067 | 1,242736 |
