# Supplementary material for: Intranasal Administration of Human MSC for Ischemic Brain Injury in the Mouse: In Vitro and In Vivo Neuroregenerative Functions
Source: PLoS One. 2014 Nov 14;9(11):e112339. doi: 10.1371/journal.pone.0112339 (PMC4232359; doi:10.1371/journal.pone.0112339)
Supplement: Table S7 — Raw data of CRT measurements shown in “ Figure 4 . Dose effect of hMSC on motor performance and lesion volume”. (Outlier detected with Grubbs test p<0.05). (DOCX) [file pone.0112339.s008.docx]

**Tabel S7**

| Sham |  | Vehicle |  | 1x10^6^ |  | 2x10^6^ |
| --- | --- | --- | --- | --- | --- | --- |
| 11,76 |  | 40,00 |  | 10,00 |  | 20,00 |
| 6,25 |  | 60,00 |  | 20,00 |  | 11,54 |
| 0,00 |  | 23,53 |  | 47,62* |  | 7,69 |
| 6,25 |  | 10,00 |  | 25,00 |  | 27,27 |
| 0,00 |  | 38,46 |  | 20,83 |  | 8,33 |
| 14,29 |  | 56,25 |  | 27,78 |  | 0,00 |
| -5,00 |  | 38,09 |  | 10,53 |  | 19,05 |
| 0,00 |  | 33,33 |  | 8,33 |  | 10,00 |
| 9,09 |  | 35,29 |  | 14,29 |  | 8,33 |
| 0,00 |  | 30,00 |  | 18,75 |  | 5,55 |
| -8,33 |  | 50,00 |  | 9,09 |  | 13,33 |
| 13,33 |  | 20,00 |  | 9,09 |  | 20,00 |
| 5,26 |  | 20,00 |  |  |  |  |
|  |  | 30,00 |  |  |  |  |
|  |  | 17,65 |  |  |  |  |
|  |  | 20,00 |  |  |  |  |
|  |  | 53,33 |  |  |  |  |
|  |  | 33,33 |  |  |  |  |
|  |  | 47,83 |  |  |  |  |
|  |  | 20,00 |  |  |  |  |
|  |  | 7,69 |  |  |  |  |
|  |  | 7,69 |  |  |  |  |
|  |  | 0,00 |  |  |  |  |
