# Supplementary material for: Intranasal Administration of Human MSC for Ischemic Brain Injury in the Mouse: In Vitro and In Vivo Neuroregenerative Functions
Source: PLoS One. 2014 Nov 14;9(11):e112339. doi: 10.1371/journal.pone.0112339 (PMC4232359; doi:10.1371/journal.pone.0112339)
Supplement: Table S8 — Raw data of MBP measurements shown in “ Figure 4 . Dose effect of hMSC on motor performance and lesion volume”. (DOCX) [file pone.0112339.s009.docx]

**Table S8**

| Sham |  | Vehicle |  | 1x10^6^ |  | 2x10^6^ |
| --- | --- | --- | --- | --- | --- | --- |
| 0,54 |  | 45,63 |  | 42,30 |  | 25,48 |
| 3,53 |  | 17,98 |  | 76,45 |  | 17,22 |
| -2,16 |  | 73,31 |  | 42,76 |  | 21,41 |
| -1,01 |  | 57,92 |  | 35,90 |  | 9,54 |
| 3,72 |  | 13,04 |  | 43,75 |  | 10,66 |
| 0,06 |  | 77,96 |  | 66,85 |  | 10,73 |
| 6,49 |  | 76,63 |  | 28,60 |  | 4,74 |
| 0,00 |  | 74,28 |  | 50,77 |  | 7,86 |
| 0,24 |  | 84,81 |  | 49,47 |  | 12,12 |
| 6,79 |  | 16,75 |  | 53,23 |  | 11,95 |
| -0,92 |  | 41,75 |  | 11,55 |  | 18,05 |
| 7,55 |  | 78,37 |  |  |  | 1,46 |
| 13,35 |  | 58,16 |  |  |  |  |
|  |  | 78,60 |  |  |  |  |
|  |  | 59,73 |  |  |  |  |
|  |  | 60,22 |  |  |  |  |
|  |  | 33,64 |  |  |  |  |
|  |  | 49,35 |  |  |  |  |
|  |  | 75,02 |  |  |  |  |
