# Supplementary material for: Intranasal Administration of Human MSC for Ischemic Brain Injury in the Mouse: In Vitro and In Vivo Neuroregenerative Functions
Source: PLoS One. 2014 Nov 14;9(11):e112339. doi: 10.1371/journal.pone.0112339 (PMC4232359; doi:10.1371/journal.pone.0112339)
Supplement: Table S9 — Raw data of MAP2 measurements shown in “ Figure 4 . Dose effect of hMSC on motor performance and lesion volume”. (DOCX) [file pone.0112339.s010.docx]

**Table S9**

| Sham | Vehicle |  | 1x10^6^ |  | 2x10^6^ |
| --- | --- | --- | --- | --- | --- |
| 3,85 | 57,91 |  | 41,25 |  | 24,11 |
| 9,30 | 60,71 |  | 45,35 |  | 13,94 |
| 3,06 | 69,41 |  | 27,81 |  | 20,51 |
| 1,46 | 43,92 |  | 43,71 |  | 10,22 |
| 3,08 | 66,36 |  | 21,21 |  | 26,87 |
| 4,16 | 16,14 |  | 30,59 |  | 22,31 |
| 7,97 | 69,59 |  | 61,11 |  | 17,96 |
| 12,74 | 19,78 |  | 26,58 |  | 9,94 |
| -1,72 | 5,68 |  | 13,16 |  | 9,58 |
| 1,05 | 35,88 |  | 42,55 |  | 19,53 |
| 10,70 | 44,42 |  | 21,25 |  | 21,53 |
| 3,79 | 40,17 |  |  |  | -2,42 |
| 1,16 | 70,09 |  |  |  |  |
|  | 51,60 |  |  |  |  |
|  | 63,95 |  |  |  |  |
|  | 41,79 |  |  |  |  |
|  | 64,16 |  |  |  |  |
|  | 36,60 |  |  |  |  |
|  | 18,08 |  |  |  |  |
|  | 25,58 |  |  |  |  |
|  | 28,33 |  |  |  |  |
