# Supplementary material for: Intranasal Administration of Human MSC for Ischemic Brain Injury in the Mouse: In Vitro and In Vivo Neuroregenerative Functions
Source: PLoS One. 2014 Nov 14;9(11):e112339. doi: 10.1371/journal.pone.0112339 (PMC4232359; doi:10.1371/journal.pone.0112339)
Supplement: Table S10 — Raw data of Iba-1+ signal measurements shown in “ Figure 5 . hMSCs reduce the activation of glial cells at 28 days after HI”. (DOCX) [file pone.0112339.s011.docx]

**Table S10**

| Sham | Vehicle | 1x10^6^ | 2x10^6^ |
| --- | --- | --- | --- |
| 137752,1 | 484862,0 | 348862,3 | 138386,2 |
| 206022,2 | 312457,7 | 421552,1 | 195393,5 |
| 114370,4 | 461644,4 | 538696,2 | 324416,8 |
| 97800,4 | 420196,8 |  |  |
