# Supplementary material for: Intranasal Administration of Human MSC for Ischemic Brain Injury in the Mouse: In Vitro and In Vivo Neuroregenerative Functions
Source: PLoS One. 2014 Nov 14;9(11):e112339. doi: 10.1371/journal.pone.0112339 (PMC4232359; doi:10.1371/journal.pone.0112339)
Supplement: Table S11 — Raw data of GFAP+ signal measurements shown in “Figure 5. hMSCs reduce the activation of glial cells at 28 days after HI”. (DOCX) [file pone.0112339.s012.docx]

**Table S11**

| Sham |  | Vehicle |  | 1x10^6^ | 2x10^6^ |
| --- | --- | --- | --- | --- | --- |
| 336418,4 |  | 414863,4 |  | 341060,3 | 258745,5 |
| 294917,1 |  | 407009,4 |  | 290828,3 | 274037,5 |
| 289612,5 |  | 467415,2 |  | 234254,1 | 308961,3 |
| 345534,1 |  | 426224,7 |  |  |  |
